# Value acquisition, value co-creation: The impact of perceived organic grocerant value on customer engagement behavior through brand trust

**DOI:** 10.3389/fpsyg.2022.990545

**Published:** 2022-10-05

**Authors:** Weiping Yu, Mingli He, Xiaoyun Han, Jun Zhou

**Affiliations:** Business School, Sichuan University, Chengdu, China

**Keywords:** organic grocerant, perceived value, customer engagement behavior, brand trust, value co-creation

## Abstract

Affected by COVID-19, there is a growing trend toward healthy lifestyles and organic food consumption. The literature on organic foods focuses on the factors that influence buying behavior. A thriving organic business requires both sustained consumption and consumer contributions beyond the purchase—customer engagement behavior. The purpose of this study is to examine the factors that may drive member customers to engage with organic grocerants. This study surveyed 280 Chinese member customers of an organic grocerant to explore how to drive customer engagement behavior. Based on value co-creation theory and the customer engagement literature, this study proposed a “value acquisition–value co-creation” framework to explore the relationship between perceived value, brand trust, and customer engagement behavior. The results show that emotional and social value can directly and effectively motivate customer engagement behavior in organic grocerants. However, consumers’ perceived quality value and price value will not directly affect customer engagement behavior but instead indirectly affect it through brand trust. Furthermore, improving the perceived value of emotion, quality and price can strengthen brand trust in organic grocerants. The study confirms that brand trust is critical to organic grocerant and customer engagement. Our findings provide a new perspective for understanding the relationship between the value customers receive from organic food consumption and value co-creation through customer engagement behavior.

## Introduction

The COVID-19 pandemic has continued to change the way people live, eat, and consume since 2019. Outside of health and environmental protection, organic food consumption has been increasing in multiples (Rana and Paul, 2017), and people prefer a healthy diet (Liu et al., 2021). From 2013 to 2020, the market size of China’s organic food industry increased from 27.98 billion yuan to 71.4 billion yuan, and China is now the largest organic food market in Asia and the fourth largest in the world (Willer et al., 2022). With the fast pace of modern life, people prefer integrated food service providers that offer healthy and nutritious foods, ready-to-eat products, meals made on site, and fresh ingredients that can be ordered and delivered to their homes (Lin et al., 2021; Mahmood et al., 2022). The “grocerant,” a new trend in the food sector, is a result of the fusion of food retail and foodservice. Organic grocerants are places that sell organic food, deliver organic food services, and offer organic living experiences (Hong and Ahn, 2021). As the new coronavirus pneumonia epidemic has swept the world, leading to the widespread use of noncontact services (Lee and Lee, 2020), organic grocerants meet the changing needs of consumers for high quality, convenience, freshness, health, and home delivery (Ham et al., 2021).

Customer engagement behavior aggregates the many ways in which customer behavior outside of trading can affect companies and is divided into four categories: co-developing behavior, influencing behavior, augmenting behavior, and mobilizing behavior (Jaakkola and Alexander, 2014). Customers not only buy products or services but also positively influence the company through non-transactional behaviors (Roy et al., 2018). While customer engagement behavior has been extensively studied in the marketing field, less research has been done on the gradually flourishing organic grocerants. Organic grocerants, compared to traditional restaurants and supermarkets, have factors such as high prices, lack of variety, attitude-purchase gaps, lack of consumer knowledge, and consumer distrust (Chekima et al., 2017; Feil et al., 2020; Hong and Ahn, 2021). The effective management of organic grocerant customers’ engagement is critical to improving corporate marketing and sales performance. It not only helps to reduce customer churn and customer switching behavior but also builds and maintains long-term customer-brand relationships and improves corporate performance (Konuk, 2019; Ng et al., 2020; Foroudi et al., 2021). Therefore, it is a challenging task for marketers to attract consumers to make contributions beyond the purchase.

Although an attitude-purchase gap exists in organic consumption, the reality is that a segment of the population is still willing to buy consistently and contribute to brands and companies beyond their purchases. Earlier research has shown that brand members are excellent business partners because they take a more active role in social interactions, word-of-mouth promotion, and product creation (Liao et al., 2017). Therefore, this study focuses on member customers who have been consuming organic food for a long time, which is more authentic and meaningful. Membership is an effective way to promote continuous organic consumption and build a deep bond with the brand (Zhang et al., 2020b). Membership is a semi-contractual relationship with a company by paying a certain amount in advance (Borle et al., 2008). Organic grocery members are entitled to certain discounts and better value-added membership services. Member community management also gives companies and members a stable platform for co-creating value together over the long-term, with customers becoming involved often (Liao et al., 2017). Research has shown that consumer perceptions of “value for money” at restaurants positively influence repeat visits, recommendations, and word of mouth (Liu and Jang, 2009). Consumer perceived value drives greater consumer engagement with brands through identification (Bhattacharya and Sen, 2003). The perceived value of member customers may exceed that of non-member customers, so member customers will bring more value to the business. Thus, member customer perceived value drives customer engagement behavior (Brodie et al., 2011), and customer engagement is a prerequisite for value co-creation (Hollebeek, 2019). Customer engagement provides motivation for value co-creation, can provide suggestions for improving products/services (Ho et al., 2020), attract potential new customers (van Doorn et al., 2010), and contributes to long-term development (Roy et al., 2018).

A growing number of businesses are utilizing digital business models, new retail transformations, and brand membership communities to actively interact with customers and produce long-term value in response to the “new normal” of COVID-19 and the harsh market rivalry (Wang et al., 2022). Value co-creation is generated in the interaction between enterprises and customers (Grönroos and Voima, 2013). Customer engagement behavior helps enterprises understand the process of value co-creation and establish long-term customer-enterprise relationships (Nkoulou Mvondo et al., 2022). The core of brand-customer management has undergone an evolutionary process of “transaction-relationship-engagement” (Pansari and Kumar, 2017; Shulga and Busser, 2020). However, the research on organic consumption is still based on the single path of “enterprises create value through products and services and customers benefit through consuming” (Hsu et al., 2019). Many studies have placed multiple perceived value dimensions on the behavioral intentions of restaurant brands (Itani et al., 2019; Zhang et al., 2021). The story of China’s fast-growing organic food market and the burgeoning organic grocerant has rarely been studied. Research on customer engagement behavior based on value co-creation theory in the context of organic grocerants is still a treasure field to be explored urgently. Starting from specific perceived values, including emotional, social, quality, and price values, this study can more clearly explore which kind of value is more effective for the value brought by consumers to organic grocerants.

Trust is a crucial factor affecting organic consumption because it is difficult to know its properties by touching and eating it (Lee and Hwang, 2016; Massey et al., 2018). Especially during the COVID-19 pandemic, consumers’ trust in restaurants and brands can influence their visit intention (Hakim et al., 2021). Point-of-sale information enhances consumer trust, which in turn bridges the intent-behavior gap between organic grocery customers (Frank and Brock, 2018). Previous studies on consumers’ trust in organic consumption tend to focus on the factors of the organic product itself, such as product certification standards (Vega-Zamora et al., 2019), environmentally friendly production (Qi et al., 2020), health, and nutrition attributes (Nguyen et al., 2019b). However, less research has been conducted on organic food service provider brands, and there is a lack of analysis of the emotional and social motivations for brand trust antecedents. Consumers’ perceived value will further affect their psychological state and behavior (Lian et al., 2016; Hsu et al., 2019). Our study returns to the essence that “what customers consume is not the product but value” (Drucker, 1954) and raises the following two research questions:

RQ1: How does the value consumers perceive from organic grocerants motivate their engagement behaviors to contribute to the brand?

RQ2: What role does brand trust play between customer perceived value and engagement behavior in organic grocerants?

This study transcends the literature on consumer purchasing behavior and explores the influence of various dimensions of organic grocerant perceived value on brand trust and engagement behavior. We conceptualize this process as value acquisition—value co-creation. The research objects are organic grocerant member customers, and the results deepen customer value theory and brand practice in the organic food service industry.

## Literature review and hypothesis development

### Organic grocerant and member customers

Grocerant offers food service and product retail in the same commercial space, making it a fun place for consumers to enjoy food, experience shopping and social interaction (Kim et al., 2019). For example, Eataly in Italy, Whole Foods and Hy-Vee in the United States, Freshippo and Super Species in China. Organic grocerants provide organic food retail, on-site organic catering services, and fresh organic ingredient delivery services to consumers. Organic grocerants are favored by consumers for offering safe, healthy, and nutritious organic food (Chekima et al., 2017; Gomiero, 2018) and for the shift in consumer values and lifestyles, such as self-care, ecological values and sustainable lifestyles (Rana and Paul, 2017). Most of the existing research on customer behavior in the organic consumption sector is related to purchasing behavior, with extensive research and reviews of customer values, attitudes, emotions, perceived values, personal norms, involvement, consumer awareness, educational background, and age (Rana and Paul, 2017). There are also empirical studies on organic business aspects such as pricing, organic labeling, product information, traceability information, membership programs, reputation, and organic certification systems (Gomiero, 2018; Hsu et al., 2019). However, less research has been performed on organic restaurants and organic grocerants.

Although consumers generally hold a positive attitude toward organic food, the inconsistency between their attitude and actual behavior has also been found by scholars (Chekima et al., 2017). Organic grocerants started using various strategies to promote sustainable consumption among consumers. Membership systems (Liao et al., 2017) and brand community management (Luo et al., 2015; Raïes et al., 2015) can effectively increase organic consumption and build brand equity. The former can increase the revenue of grocerants, while the latter can promote value co-creation between customers and grocerant brands (Tajvidi et al., 2020; Veloutsou and Black, 2020).

Unlike community-supported agriculture (Cristiano, 2021), organic grocerant membership is a semi-contractual relationship established by organic grocerants and customers. Organic customers prepay amounts to obtain better service and support the development of the grocerant (Singh and Jain, 2010). The fixed consumption and quota of the members are relatively high (Zhang et al., 2020b), and the cooperative relationship with the grocerant brand is stable (Bruneau et al., 2018). Furthermore, with the development of information technology, organic brand community management is more convenient, and customer interaction is more abundant (Ahmad and Zhang, 2020). Organic grocerants bring more value to member customers, and customers will spontaneously share good things, help others, and provide suggestions to grocerants (Ahmad and Zhang, 2020; Yu et al., 2021). The value that customers bring to brands becomes diverse and valuable.

### Value co-creation theory

Value co-creation is considered to be a key principle of service-dominant logic (Vargo and Lusch, 2004; Zaborek and Mazur, 2019). Companies can provide products or services to create value, and customers create value by using the product and interacting (e.g., sharing expectations and experiences, etc.) after receiving the value proposition, and customers are always cocreators of value (Hong et al., 2021). Brands and customers cocreate value through relationship interaction and resource integration (Vargo and Lusch, 2008). Both customers and companies are pursuers of value maximization. Customers can engage in product design, production, delivery, and consumption (Vargo and Lusch, 2008) and change from passive value recipients to active creators (Galvagno and Dalli, 2014). The power of the market is increasingly shifting to customers (Itani et al., 2019). Highly engaged customers become the source of brand value (Chen et al., 2022). Cocreating value with customers has become a driver of business performance and continues to generate revenue for the business (Zaborek and Mazur, 2019).

Brands can gain sustainable competitive advantage through customer engagement behavior (Pansari and Kumar, 2017) and drive value from customer to enterprise (Kumar et al., 2010). In the organic food market, customers are not only consuming brands’ products but also marketing them (Yu et al., 2021). The co-creation of experiences between customers and restaurant brands positively affects customer brand engagement, brand attachment, and customer satisfaction (Hussain et al., 2021). Customer perceived value, trust, and loyalty are essential antecedents to customer engagement (van Doorn et al., 2010), and engagement is a critical mechanism to develop co-creation ability (Harrigan et al., 2021). However, scholars and practitioners lack a complete understanding of how customer value perception affects customer engagement behavior. Research on the engagement behavior of organic grocerant customers is still in its infancy. This study will focus on member customers and explore the process of value perception and value creation between consumers and brands based on value co-creation theory.

### Perceived value

Understanding consumers’ perceived value of organic grocerants is the basis for understanding consumers’ expectations, which is helpful for brands to develop more accurate marketing strategies to meet consumers’ needs (Curvelo et al., 2019). Consumers’ perceived value of organic grocerants is related to the organic food they sell, the organic diet they provide, and the organic events they organize. The perceived value of organic food is not only related to the nutritional content, safety, and taste of the product (Rana and Paul, 2017; Nguyen et al., 2019a). It is also about the emotional connection (Watanabe et al., 2020) and social identification (Du et al., 2017) that can be made in the consumption of organic food and sometimes the willingness to pay a higher price (Aschemann-Witzel and Zielke, 2017).

The perceived value of organic grocerants has multiple value dimensions; the quality and price of organic grocerants, the emotional experience, and the social benefits of organic grocerants have received more attention in academic research and enterprise practice. Sweeney and Soutar (2001) integrated the meaning of customer value into four dimensions of quality, price, emotional, and social values based on previous studies and developed a perceived value measurement scale widely used in subsequent studies. Later, Khan and Mohsin (2017) pointed out that price and social value positively influence Pakistani consumers’ green product choice behavior. Kim et al. (2019) confirmed that brand prestige affects customer loyalty through quality, price, and social value. Regarding organic consumers in Brazil, functional and emotional value can affect consumer trust, and only emotional value can stimulate purchase intention (Watanabe et al., 2020). It can be seen from the above viewpoints that the four dimensions of the perceived value of organic grocerants have different roles in different countries and objects (van Doorn et al., 2010; France et al., 2018). The perceived value of organic grocerants was operationally defined as follows:

The quality value is derived from the product’s utility and desired performance (Sweeney and Soutar, 2001; Watanabe et al., 2020). Regarding organic grocerants, quality value refers to the perceived utility of safety, nutritional health, quality standards, and stability obtained by customers from purchasing (eating) organic food in organic grocerants (Rana and Paul, 2017).

Price value refers to the perceived utility that customers get from the organic grocerant’s purchase (catering), such as reasonable pricing, value for money, and exceptional product/service relative price. Organic food and dining are usually more expensive due to their healthier, environmentally friendly, pesticide-free features, and their high price further highlights these qualities (Mondelaers et al., 2009).

Emotional values are related to the emotions that consumers feel when purchasing products or experiencing services at organic grocerants (Sweeney and Soutar, 2001; Holbrook, 2006). Previous studies have also shown that feelings of relaxation and happiness are predominant for organic food consumption (Lee and Yun, 2015; Apaolaza et al., 2018). Hedonic experience and food health are important factors that influence the intention to revisit organic grocerants (Yoo et al., 2020). Cognitive evaluation and emotional experience are the main factors that affect consumers’ purchasing decisions (Lindeman and Verkasalo, 2005).

Social value is related to the social acceptance of a specific reference group (Watanabe et al., 2020). It refers to the perceived utility customers get from the consumption and experience of organic grocerants, such as accepting, leaving a good impression, improving others’ views, and winning social identity. Consumers’ cognition of organic food or organic restaurants interacts with the processes related to their social identity (Hong and Ahn, 2021).

### Brand trust

The perceived potential benefits of organic foods (health nutrition, happiness, social acceptance, and value for money) may have a positive impact on consumers’ internal psychology (brand trust) and behavior (customer engagement). Brand trust is defined as consumers’ willingness to recognize a grocerant brand under risky situations based on positive expectations of the grocerant’s brand quality, behavioral intentions, and ability to deliver on commitments (Munuera-Aleman et al., 2003; Grayson et al., 2008). Numerous studies have confirmed that consumers are skeptical of green product claims (Chen and Chang, 2013), and organic food and organic restaurants are no exception (Jäger and Weber, 2020). Trust has been identified as a prerequisite for establishing an organic market (Lee et al., 2019). Because most consumers do not have the expertise to distinguish organic food from its emphasis on pesticide-free, cleaner production (Hartmann et al., 2018). Therefore, consumers are more likely to consume and purchase from trusted restaurant brands or grocery brands (Yu et al., 2021). A good brand reputation will influence consumers’ attitudes and behavior toward organic consumption (Ryan and Casidy, 2018). When consumers trust a grocerant brand, they believe it offers high-quality organic food, reducing uncertainties (Han et al., 2015). Trust has become an urgent part of building a positive and lasting relationship between brands and consumers.

Many investigations have revealed the positive relationship between consumers’ perception of quality and trust in organic food and organic restaurants (Konuk, 2018; Lee et al., 2019). When consumers perceive safety and quality, they will further trust the brand and buy it (Vega-Zamora et al., 2019). Therefore:

*H1*: The quality value of organic grocerants has a positive influence on customers’ brand trust.

Price is often cited as the main factor limiting organic consumption, which is related to perceived economic costs and benefits (Lee et al., 2019). Consumers’ perceived price value can positively influence purchasing behavior and trust (Aschemann-Witzel and Zielke, 2017). Concurrently, there is also evidence that consumers are suspicious of organic food when the price is too low (Konuk, 2019). Therefore:

*H2*: The price value of organic grocerants has a positive influence on customers’ brand trust.

Brand trust also derives from consumers’ emotional attachment. Lee and Yun (2015) investigated consumers’ motivation to buy organic food and confirmed that the combination of hedonic and emotional value positively influences consumers’ purchase intention. Watanabe et al. (2020) found that only emotional value can determine consumers’ trust in organic food and discretion to buy it. The feel-good effect of buying organic food significantly promotes consumer attitudes and behavior. Therefore, we propose the following hypothesis:

*H3*: The emotional value of organic grocerants has a positive influence on customers’ brand trust.

Individuals tend to express themselves in consistent ways with their self-identity (Burke and Reitzes, 1991). Moreover, Chinese consumers love brands with a sense of social responsibility (Dang et al., 2020), and spending on such brands can help them leave a good impression in social groups (Du et al., 2017). Organic grocerant and organic food enterprises are practitioners of corporate social responsibility (Yu et al., 2021). The perceived social value of consumers may promote their trust in brands (Ladwein and Romero, 2021). Therefore, we posit:

*H4*: The social value of organic grocerants has a positive influence on customers’ brand trust.

### Customer engagement behavior

Customer engagement behavior stems from motivation-driven behavior, which goes beyond transactions and may be precisely defined as a customer’s behavioral manifestations with a brand or firm focus beyond purchase (van Doorn et al., 2010). Jaakkola and Alexander (2014) and Roy et al. (2018) categorize customer engagement behavior as co-developing behavior (helping a firm’s development process), augmenting behavior (augmenting an offering), influencing behavior (affecting or changing other customers’ perceptions, behavior, and knowledge), and mobilizing behavior (mobilizing other stakeholders’ behaviors toward the organization). Value co-creation is generated in stakeholder interaction through resource integration (Galvagno and Dalli, 2014). Customer engagement behavior promotes the value flow between the customer and the brand. It influences the other stakeholders’ value co-creation by indirectly affecting their perception, knowledge, preference, expectation, or action on the company or the product (van Doorn et al., 2010). The value that customers obtain or perceive from consumption will urge them to create benefits for enterprises through engagement behaviors, summarized as the “value gets, and value gives” framework (Itani et al., 2019).

Customer engagement behavior can effectively improve brand reputation (Algharabat et al., 2020) and loyalty, which are the two competitive advantages organic grocerants need. Therefore, organic grocerant brands should pay attention to customer engagement to bring long-term development to enterprises (Yu et al., 2021). Engaged customers can directly contribute to firms’ performance (Kumar et al., 2010; Cheung et al., 2020). Customers can indirectly contribute to a brand’s business success through recommendations, social media conversations, and feedback and suggestions (Pansari and Kumar, 2017). Especially with social networks, it amplifies the social impact of consumers on the focus brand (Algharabat et al., 2020; Itani et al., 2020); promotes positive relationships between consumers and brands (Banyte and Dovaliene, 2014); and establishes a value co-creation system among enterprises, customers, and stakeholders (Agrawal et al., 2015).

Previous studies have shown that the value perceived by customers in consumption or experience will affect consumers’ choice, evaluation, purchase, trust, satisfaction, and further their advocacy behavior (Kim et al., 2019; Sharma and Klein, 2020). Roy et al. (2018) also confirmed that customer engagement behavior is influenced by trust and value in use. Numerous empirical studies have shown that customer engagement behavior increases when they perceive the excellent quality of products and services (Itani et al., 2019) or have a positive emotional attachment (Mingione et al., 2020). It is also aroused when the customer feels value for money and a reasonable assessment of the premium paid for the environmental and health benefits of organic food (Konuk, 2018; Septianto et al., 2019) or meets a comparative social need from the exchange relationship and gains symbolic value (Wang et al., 2018; Wongkitrungrueng and Assarut, 2020).

Based on the previous discussion on the potential quality, price, emotional, and social value of organic food, we expect to see the following relationship between consumer perceived value and customer engagement behavior:

*H5*: The quality value of organic grocerants has a positive influence on customer engagement behavior.

*H6*: The price value of organic grocerants has a positive influence on customer engagement behavior.

*H7*: The emotional value of organic grocerant has a positive influence on customer engagement behavior.

*H8*: The social value of organic grocerants has a positive influence on customer engagement behavior.

Customers who feel more confident in their suppliers and service providers are more likely to show their engagement behaviors van Doorn et al. (2010) and Pansari and Kumar (2017) identified trust as an essential antecedent of customer engagement behavior. Nuttavuthisit and Thøgersen (2017) demonstrated that customer trust is a decisive prerequisite for building a market for trust goods such as organic food. Pandey and Khare (2017) suggested that customer trust in organic food retailers is based on perceived food quality, service quality, and fair price. Many scholars have confirmed the positive effect of trust on customer engagement behavior in different contexts (Chuah et al., 2020; Li et al., 2020). Accordingly, this paper proposes the following hypothesis:

*H9* Customers’ brand trust has a positive influence on customer engagement behavior.

**Figure 1:**
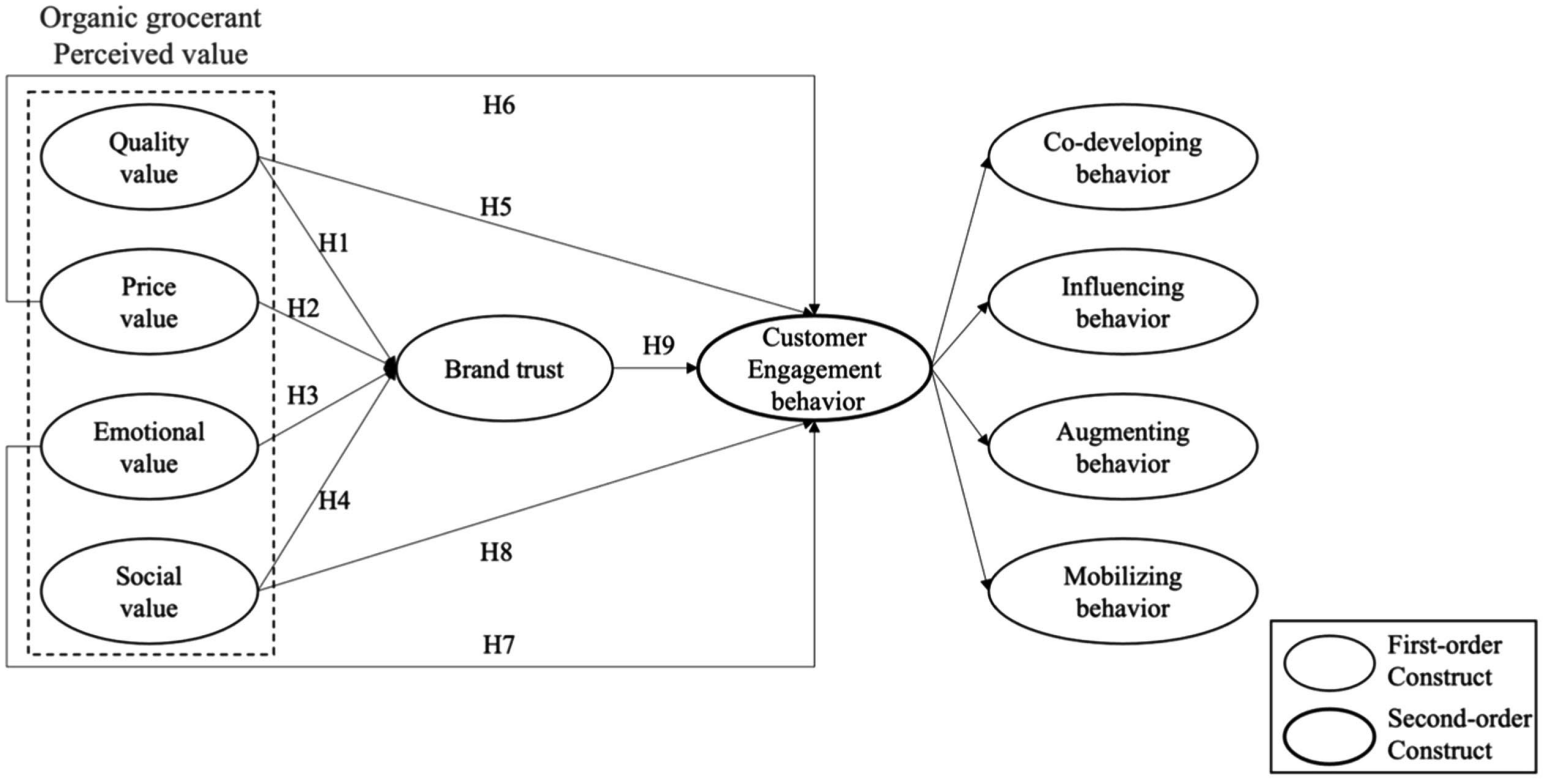
Research model.

Based on previous research, we built the research model shown in Figure 1.

## Methodology

### Questionnaire and measures

The questionnaire survey was used to conduct empirical research in this study, answered in self-reports. The initial questionnaire was constructed in English, translated into Chinese, and translated by two doctoral students in marketing with study abroad experience. The marketing professor made a final check. The questionnaire design is divided into two parts. The first part is the basic information. The second part investigates the variable measurement involved in the model. The results are only relevant to member customers who already had a semi-contractual relationship with an organic grocerant brand. Respondents have an authentic experience of the continuous consumption of organic grocerants. Where non-purchasers stand is not covered by the results of this study. A convenience sample of 33 member customers and five marketing doctoral students was pretested to see potential problems with process, clarity, or understanding. This has led to some minor changes.

All measurements were based on previous studies and adjusted for the organic grocerant context. The scale of perceived value refers to the research of Sweeney and Soutar (2001), which contains four dimensions of perceived value. The measurement of brand trust is based on the firm-specific trust scale of Grayson et al. (2008). Customer engagement behavior contains four sub-dimensions derived from the scale developed by Roy et al. (2018). All the items were measured using a 5-point Likert scale.

### Data collection

The data came from Sichuan Province, China. The samples were from M’s customers, one of the most famous organic grocerants in the province, running for 13 years. The questionnaire was distributed both online (*via* the professional online questionnaire survey website “Sojump”)^1^ and offline (the business premises of M brand). We collected data from a total of 280 respondents, all of whom are member customers of M brand who have repeatedly consumed organic food. Of this number, the majority of respondents were female (*n* = 182; 65%), aged between 30 and 50 (*n* = 217; 77.5%), and had a bachelor’s degree and above (*n* = 231, 82.5%). Among these customers, 95 members (33.9%) pre-deposit less than 5 thousand yuan, 109 members (38.9%) 5–20 thousand yuan, 64 members (22.9%) 20–50 thousand yuan, and 12 members (4.3%) more than 50 thousand yuan. Since the survey objects are all member customers and the pre-deposit amount is between 5,000 and 50,000 yuan, this study does not consider purchase intention.

### Data analysis

Data analysis was conducted using a two-step strategy of structural equation modeling (SEM). First, confirmatory factor analysis (CFA) was performed to investigate the validity and reliability of the scale. Second, SEM was used to test the research hypothesis and the mediating effect.

## Results

### Reliability and validity

The reliability test of each potential variable is required before testing the relationship between variables. Factor loadings are checked against the recommended threshold of 0.6 to assess the reliability of each item, and one of brand trust is removed from the analysis. The results are shown in Table 1. The final factor loading for each item exceeds 0.7, indicating adequate internal reliability (Chin, 1998). Meanwhile, Cronbach’s alpha and composite reliability (CR) scores of all variables were higher than 0.8 (Hair et al., 2010), indicating that the scale had sufficient internal consistency.

**Table 1:** CFA results in the survey.

Item	Loading	Cronbach’s α	CR	AVE
Quality value				
“M” organic grocerant’s food and service have consistent quality	0.864	0.92	0.92	0.742
“M” Organic grocerant’s food and meal are well-made	0.869			
“M” Organic grocerant’s food and service have an acceptable standard of quality	0.866			
“M” Organic grocerant’s food and service would perform consistently	0.847			
Price value				
“M” Organic grocerant is reasonably priced	0.851	0.903	0.906	0.763
“M” Organic grocerant offers value for money	0.925			
“M” Organic grocerant has a good product or service for the price	0.842			
Emotional value				
“M” Organic grocerant is one that I would enjoy	0.864	0.918	0.918	0.738
“M” Organic grocerant would make me want to visit	0.854			
“M” Organic grocerant is one place that I would feel relaxed	0.826			
Purchase food or services from “M” organic grocerant would make me feel good	0.891			
Social value				
Consumption in “M” organic grocerant will help me to feel acceptable	0.876	0.944	0.944	0.808
Consumption in “M” organic grocerant will affect the way that I am perceived	0.885			
Consumption in “M” organic grocerant will make a positive impression on other people	0.904			
Consumption in “M” organic grocerant will help me gain social approval	0.93			
Brand trust				
I can count on [Brand] to consider how its actions will affect customers like me	0.714	0.86	0.862	0.61
If I were to have any problems, [Brand] will be ready and willing to offer me assistance and support	0.769			
When making decisions about its policies, [Brand] is concerned about customers like me	0.797			
I can count on [Brand] to be sincere in its communication	0.838			
Co-developing behavior				
I proactively communicate with [Brand] about potential product/service-related problems	0.893	0.93	0.932	0.821
I make constructive suggestions to [Brand] about how to improve its products/services	0.943			
I let [Brand] know of ways that can better serve my needs	0.881			
Influencing behavior				
I said positive things about [Brand] and its employees to others	0.813	0.902	0.904	0.759
I recommend [Brand]and its employees to others	0.912			
I encourage friends and relatives to use [Brand] in future	0.885			
Augmenting behavior				
I post photographs of my stay at [Brand] l on social media	0.924	0.934	0.937	0.79
I would write blogs about my positive experience at [Brand]	0.955			
The [Brand] provides opportunities to share my experience with others *via* social media	0.907			
I engage in forwarding the promotions offered by the [Brand] to others	0.755			
Mobilizing behavior				
I assist other customers if they need my help	0.87	0.928	0.928	0.72
I give advice to other customers regarding the products/services of the [Brand]	0.891			
I teach other customers to use products/services correctly	0.899			
I am willing to stand to protect the reputation of the [Brand]	0.763			
I am willing to clarify other customers or outsiders misunderstanding regarding the [Brand]	0.812			

The convergent validity of the scale was tested by average variance extracted (AVE). The AVE of the nine variables ranged from 0.61 to 0.821, meeting the minimum standard of 0.5 (MacKenzie, 2011), proving great convergent validity.

The discriminant validity of the scale refers to the degree of difference between constructs. The test method is to compare the correlation coefficient between variables and the square root of AVE. If the former is less than the latter, it means that the discriminant validity of the variable is acceptable. The minimum AVE square root value is 0.781, greater than the maximum correlation coefficient of 0.759 in Table 2.

**Table 2:** Correlations of the constructs.

Construct	Mean	SD	QV	PV	EV	SV	BT	CDB	IB	AB	MB
QV	3.99	0.759	**0.861**								
PV	3.57	0.79	0.620**	**0.873**							
EV	3.958	0.698	0.756**	0.707**	**0.859**						
SV	3.278	0.969	0.468**	0.449**	0.507**	**0.899**					
BT	3.826	0.67	0.663**	0.639**	0.694**	0.416**	**0.781**				
CDB	3.79	0.84	0.521**	0.486**	0.581**	0.405**	0.627**	**0.906**			
IB	3.86	0.781	0.552**	0.583**	0.657**	0.394**	0.617**	0.736**	**0.871**		
AB	3.42	0.927	0.495**	0.514**	0.516**	0.432**	0.498**	0.661**	0.759**	**0.889**	
MB	3.65	0.771	0.507**	0.560**	0.640**	0.474**	0.611**	0.696**	0.731**	0.682**	**0.849**

Values on the diagonal bold represent the square root of the average variance extracted, while the off-diagonals are correlations, SD, standard deviation. QV, quality value; PV, price value; EV, emotional value; SV, social value; BT, brand trust; CDB, co-developing behavior; IB, influencing behavior; AB, augmenting behavior; MB, mobilizing behavior. **p < 0.01.

### Structural equation model

A structural equation model was built according to Figure 1. The proposed hypothesis is tested by the SEM of maximum likelihood estimation. The fitting index of the structural equation model reached the good standard (CMIN/DF = 2.17 < 3, CFI = 0.903 > 0.9, IFI = 0.904 > 0.9, TLI = 0.904 > 0.9, NFI = 0.891 > 0.8, RMR = 0.049 < 0.05 RMSEA = 0.078 < 0.08) (Bagozzi and Yi, 1988; Browne and Cudeck, 1992).

### Testing the hypotheses

**Figure 2:**
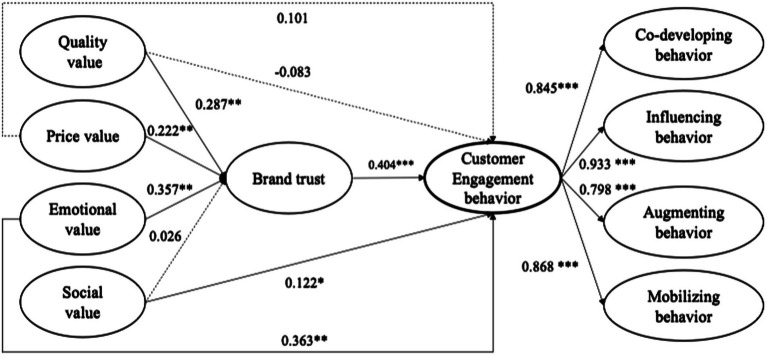
Results of the path analysis of the research model. ***p < 0.001, **p < 0.01, *p < 0.05.

The results of the structural equation model are presented in Figure 2. For simplicity, we indicate the figures’ significant paths with solid lines and the insignificant paths with dotted lines. Table 3 summarizes all path coefficients and hypothetical results.

**Table 3:** Results of hypothesis testing.

Hypotheses	Structural paths	Non-standardized coefficients		Standardized coefficients		*P*	Results
		*b*	SE	β	*t* value		
H1	QV-BT	0.275	0.091	0.287**	3.014	0.003	Supported
H2	PV-ST	0.199	0.071	0.222**	2.795	0.005	Supported
H3	EV-BT	0.338	0.11	0.357**	3.082	0.002	Supported
H4	SV-BT	0.018	0.037	0.026	0.482	0.63	Not supported
H5	QV-CEB	−0.086	0.1	−0.083	−0.863	0.388	Not supported
H6	PV-CEB	0.097	0.076	0.101	1.273	0.203	Not supported
H7	EV-CEB	0.368	0.124	0.363**	2.974	0.003	Supported
H8	SV-CEB	0.09	0.039	0.122*	2.281	0.023	Supported
H9	BT-CEB	0.434	0.101	0.404***	4.301	***	Supported

QV, quality value; PV, price value; EV, emotional value; SV, social value; BT, brand trust; CEB, customer engagement behavior. ***p < 0.001, **p < 0.01, *p < 0.05.

On the hypothesis test, it has been observed that customer perceived organic grocerant quality value (β = 0.287, *p* = 0.003 < 0.01), emotional value (β = 0.357, *p* = 0.002 < 0.01) and price value (β = 0.222, *p* = 0.005 < 0.01) are positively associated with brand trust, providing support for H1, H2, and H3. Among them, we find that emotional value has the greatest impact on the brand trust of organic grocerant consumers. However, social value does not affect customer brand trust (*t* = 0.482), so H4 is not supported.

Customer engagement behavior is a second-order construct, including co-developing behavior, augmenting an offering, influencing behavior, and mobilizing behavior (Roy et al., 2018). The scale showed adequate explanatory power for the four behavior types (see Figure 2). Regarding the impact of perceived value on customer engagement behavior, we find that emotional value (β = 0.363, *p* = 0.003 < 0.01) and social value (β = 0.122, *p* = 0.023 < 0.05) have significant effects on customer engagement behavior, supporting H7 and H8. However, neither quality value (*t* = −0.863) nor price value (*t* = 1.273) significantly influences customer engagement behavior, which means H5 and H6 are not confirmed. As predicted, brand trust significantly impacts customer engagement behavior (β = 0.404, *p* < 0.001); thus, H9 is supported.

### Mediation analyses

Although organic grocerants’ quality and price value from the existing path do not directly impact customer engagement behavior, we infer that there may be a mediating effect (Hayes, 2009). Therefore, we conduct further analysis to explore the possible role of brand trust in the relationship between four customers’ perceived value and engagement behavior. Preacher and Hayes (2008) proposed the bootstrapping procedure with 5,000 samples to construct and test (bias-corrected and percentile) confidence intervals for mediating effects. The results of indirect and direct effects are shown in Table 4.

**Table 4:** Hypothesis testing results of mediating effects.

Mediating effects	Path	Coefficient	SE	Bias-corrected 95% CI		Percentile 95% CI		Results
				Lower	Upper	Lower	Upper	
IE	QV-BT-CEB	0.118**	0.057	0.036	0.268	0.028	0.25	Full mediation
DE	QV’-CEB’	−0.092	0.097	−0.285	0.099	−0.286	0.098	
IE	PV-BT-CEB	0.085**	0.041	0.023	0.19	0.015	0.175	Full mediation
DE	PV’-CEB’	0.087	0.082	−0.081	0.239	−0.076	0.249	
IE	EV-BT-CEB	0.145**	0.069	0.043	0.327	0.034	0.301	Partial mediation
DE	EV’-CEB’	0.365**	0.137	0.11	0.648	0.101	0.636	
IE	SV-BT-CEB	0.008	0.016	−0.021	0.045	−0.023	0.042	No mediating effect
DE	SV’-CEB’	0.087*	0.041	0.005	0.166	0.009	0.171	

QV, quality value; PV, price value; EV, emotional value; SV, social value; BT, brand trust; CEB, customer engagement behavior; IE, indirect effect; DE, direct effect. **p < 0.01, *p < 0.05.

As shown in Table 4, quality value (β = 0.118; *p* = 0.002 < 0.01) and price value (β = 0.085; *p* = 0.008 < 0.01) have a significant indirect impact on customer engagement behavior through brand trust; within the 95% confidence interval, zero is not included between the upper and lower confidence intervals of bias-corrected and percentile (Shrout and Bolger, 2002). Simultaneously, when brand trust is controlled, quality value (bias-corrected CI = −0.285 to 0.099, included 0) and price value (bias-corrected CI = −0.081 to 0.239, included 0) have no direct effect on customer engagement behavior, which proves that brand trust plays a complete mediating role in this path. For emotional value, which has a greater direct influence (β = 0.365; *p* = 0.005 < 0.01) on customer engagement behavior, there was found to be an indirect effect *via* brand trust (β = 0.145; *p* = 0.003 < 0.01), which partially mediates the effect of customer perceived emotional value on customer engagement behavior. Social value has a direct effect on customer engagement behavior (β = 0.095; *p* = 0.024 < 0.05), but brand trust has no mediating effect (bias-corrected CI = −0.021 to 0.045, included 0).

## Discussion

Based on value co-creation theory and the perceived value perspective, this study explores the mechanisms that influence the formation of customer engagement behaviors of member customers toward organic grocerants. Previous research on organic grocerants and organic food has focused on consumer behavior (e.g., purchase behavior and repurchase behavior). Few studies have focused on member customers who have been consuming organic food for a long time. We wonder whether member consumers’ perceived value of organic grocerants will drive their brand trust and customer engagement behavior. The results show different mechanisms by which organic grocerant quality, price, emotional, and social values are associated with customer brand trust and engagement behavior.

First, quality, price, and emotional value directly affect brand trust, while social value does not. This suggests that having member customers experience sufficient quality value, price value, and emotional value during the consumer journey in organic grocery stores will enhance brand trust in organic grocerants. This is in line with Sankaran and Chakraborty's (2022) findings. In particular, the impact of emotional value exceeds even quality and price. Previous research has also highlighted the importance of emotions in explaining consumer behavioral intentions in environments such as retail stores, shopping centers, hotels, and restaurants (McGowan et al., 2017). The reason why social values do not influence brand trust may be explained by the fact that the subjects of the study are member customers of organic grocers and are already members of that group. The social value of consuming organic food is no longer attractive to them (Kushwah et al., 2019). It also validates previous studies that found consumers’ perceived social value from organic food does not effectively influence trust (Hansen et al., 2018; Watanabe et al., 2020).

Second, emotional and social values directly influence customer engagement behavior. It has been established that customer perceived value positively influences customer engagement behavior (Itani et al., 2019; Xie et al., 2022). Quality and price values seem to be tangible and functional, while emotional and social values are more intangible and experiential (McGowan et al., 2017). Tangible value perceptions stimulate trust and purchase behavior, while intangible value perceptions are key to stimulating non-transactional behavior. Based on social identity theory, consumer perceived value can drive consumers to be more engaged with the brand through identity (Bhattacharya and Sen, 2003). Organic grocerant members are characterized by a preference for healthy and sustainable lifestyles, and this group has a natural social and emotional identity (Johnson et al., 2012). Therefore, only emotional and social values directly influence customer engagement behavior.

Emotional value directly influences customer engagement behavior, suggesting that pleasure and happiness are very important in organic grocerant consumption. The positive experience and the pleasure of expected benefits can lead to positive consumer attitudes and behavioral feedback to the brand or company Positive experiences and the pleasure of anticipated benefits can lead to positive consumer attitudes (Watanabe et al., 2020) and behavioral feedback to the brand or company (Tajvidi et al., 2021). Existing studies have also confirmed that emotional value is the pillar of consumer and brand value co-creation (Mingione et al., 2020). Social value is related to consumers’ perceived self-image and social acceptance from a particular reference group. Customers’ self-efficacy (Oh and Syn, 2015), self-image drive (Burnasheva and Suh, 2021), and social identity (An et al., 2019) are reflected in the pursuit of social values that have been shown to have a significant impact on electronic word-of-mouth, referrals and advocacy. These findings provide more empirical evidence for the impact of social value on customer engagement behavior (Choi and Kandampully, 2019; Zhang et al., 2020a).

Finally, this study verifies the mediating role of brand trust in perceived value and customer engagement behavior. Specifically, when consumers perceive organic grocerant products and food as consistent in quality and value for money, it promotes customer trust in the brand, which in turn influences customer engagement behavior. The likely explanation is that member customers themselves have high health expectations of organic grocerants, and they take it for granted that organic grocerants are supposed to provide higher quality products and services and a more nutritious diet (Gomiero, 2018; Konuk, 2019). Meanwhile, organic products are typically trustworthy goods, and trust can offset the risk factor in the consumer experience and mitigate uncertainty, thereby reducing transaction costs (Grayson et al., 2008). This study also confirms the importance of trust in customer engagement behavior (Chuah et al., 2020), especially that consumers’ confidence in a brand can motivate them to do something beneficial.

## Implications and limitations

### Theoretical implications

By examining the relationship between the customer’s perceived value of organic grocerants and customer engagement behaviors that encourage customers to become marketers of organic grocerants, our research significantly contributes to the literature on food service and marketing. Although customer engagement has been widely researched, it is easily overlooked in the food service and hospitality industries. The use of price, quality, emotional, and social dimensions of customer perceived value as drivers of customer engagement behavior expands how the perceived value literature and value co-creation theory are used in the hospitality literature. The basic contribution of this study is to provide theoretical foundations and empirical evidence that make incremental contributions to existing knowledge.

First, this study reveals a novel model of organic consumer behavior—value acquisition-value creation. This framework deepens the study of Itani et al. (2019). The value customers receive from the organization will drive them to create greater value and benefits through customer engagement behaviors.

Second, we increased the knowledge on perceived value and customer engagement in the organic grocerant literature. The impact on customer engagement behavior was explored in terms of four dimensions of organic grocerant perceived value (quality, price, emotional, and social). This distinguishes it from previous studies on organic grocerant perceived value (Yoo et al., 2020; Jeon and Yoo, 2021). In addition, this study identifies the important role of emotional and social value in customer engagement, further expanding the understanding of the drivers of customer engagement behavior.

Third, this study extends the knowledge of brand trust in the organic grocerant consumption scenario. This study found differences in the impact of perceived value on trust between Chinese consumers and consumers in other countries (Canada, Brazil, and Malaysia) (Lian et al., 2016; Persaud and Schillo, 2017; Watanabe et al., 2020). Moreover, quality and price value influence customer engagement behavior through brand trust. It is further emphasized that trust is not only crucial for organic consumption (Vega-Zamora et al., 2019) but also an important prerequisite for customer engagement (Roy et al., 2018).

Last, the focus of this study is on member customers of organic grocerants who consume consistently, not on potential buyers whose words and actions were previously inconsistent (Chekima et al., 2017). The semi-contractual relationship represented by membership further promoted the organic industry (Singh and Jain, 2010; Yu et al., 2021). It also confirms that the long-term growth of organic consumption needs to focus on those critical consumers who will bring more value to the enterprise.

### Practical implications

First, this study provides new ways for organic grocerant managers and marketers to effectively manage customer engagement. Focusing on marketing and management strategies that give customers more value and build trust in the brand is a good way to engage customers. This is a long-term process, as brand trust takes time to achieve. Managers need to focus on customer value when selecting organic products, pricing services, and planning activities. Meanwhile, managers should segment customers and find different value types for personalized marketing.

Second, brand trust can be built and maintained in a variety of ways to make customers feel healthy and safe. Grocerants should strictly control access standards for organic food and the production of organic catering and make consumers feel healthy and safe through traceability systems, secure and transparent supply chains, and open kitchens (Zhang et al., 2021; Chuah et al., 2022). Grocerants also need to make consumers feel value for money, and prices that are too high or too low will become an obstacle for consumers (Aschemann-Witzel and Zielke, 2017). Quality value and price value are the basis of consumers’ trust in the brand, and they will do more beneficial to the brand because of faith.

Third, organic grocerants can create an enjoyable experience for consumers by conducting entertainment activities and cause-related marketing. Grocerants can enhance the emotional experience of their customers by offering high-quality organic food and organic dining, interesting organic knowledge sharing and rich organic experience activities (e.g., baking). Social value is second only to emotional value in its influence on engagement behavior. Organic grocerants should gain consumer recognition by consistently providing high-quality organic food and service and building a trustworthy brand image. These will also encourage consumers to recommend in the circle of friends, make a good impression, and give feedback to brands (Chuah et al., 2020).

Finally, this research provides recommendations for companies’ customer membership management strategies. Member customers contribute directly to the brand through purchase and contribute indirectly to the brand through engagement behavior. Through social media and brand communities, enterprises communicate closely with member customers and make intelligent, customized production based on member interaction and feedback (Algharabat et al., 2020). Customers can rely on social networks to carry out information searches, communicate with brands, share opinions, and recommend good things through social media, all of which make the value creation of C2B possible (Liao et al., 2017). Membership will be a business model for organic grocerants to further flourish in the broader Chinese market (Zhang et al., 2020b) but also around the world.

### Limitations and future research

This study has specific theoretical and practical significance and has limitations and the potential to expand in the future. First, the research object of this paper can be further expanded and subdivided. Our research mainly focuses on the member customers of one grocerant brand, which can be used as a starting point for the subsequent investigation compared with ordinary organic consumers or even expanded to multiple countries. Second, this study did not include other variables that might influence consumer engagement. In the future, variables such as value awareness, community support, and brand preference can be considered moderators. Finally, due to the difficulty of obtaining data from organic grocers, this study has not yet refined the perceived value of organic grocerants to the product or service level and compared the differences in the impact of perceived value on brand trust and engagement behavior. Therefore, it is necessary to take this as the starting point for subsequent studies to explore new research opportunities.

## Data availability statement

The raw data supporting the conclusions of this article will be made available by the authors, without undue reservation. Further inquiries can be directed to the corresponding author.

## Author contributions

WY: project administration, supervision, and funding acquisition. MH: conceptualization, formal analysis, investigation, data curation, writing–original draft, writing–review and editing, and visualization. XH: conceptualization, formal analysis, investigation, original draft, writing–review and editing, and visualization. JZ: data curation, investigation, and writing–review and editing. All authors contributed to the article and approved the submitted version.

## Funding

This work was supported by the National Office for Philosophy and Social Sciences of China (Grant No. 18AGL010).

## Conflict of interest

The authors declare that the research was conducted in the absence of any commercial or financial relationships that could be construed as a potential conflict of interest.

## Publisher’s note

All claims expressed in this article are solely those of the authors and do not necessarily represent those of their affiliated organizations, or those of the publisher, the editors and the reviewers. Any product that may be evaluated in this article, or claim that may be made by its manufacturer, is not guaranteed or endorsed by the publisher.

## Footnotes

^1^ www.sojump.com
